# Prolactinomas Resistant to Treatment With Dopamine Agonists: Long-Term Follow-Up of Six Cases

**DOI:** 10.3389/fendo.2018.00625

**Published:** 2018-11-13

**Authors:** Maria de Fátima de Magalhães Gonzaga, Lucas Faria de Castro, Luciana Ansaneli Naves, José Luiz Mendonça, Benicio Oton de Lima, Iruena Kessler, Luiz Augusto Casulari

**Affiliations:** ^1^Endocrinology Service, Brasilia University Hospital, Brasília, Brazil; ^2^Clinic of Neurology and Endocrinology, Brasília, Brazil; ^3^Medical Clinic Service, Brasilia University Hospital, Brasília, Brazil; ^4^Clinic of Radiology Vila Rica, Brasília, Brazil; ^5^Neurosurgery Unit, Hospital de Base do Distrito Federal, Brasília, Brazil; ^6^Institute of Cardiology of the Federal District, University Foundation of Cardiology, Brasília, Brazil; ^7^University of Brasilia, Brasília, Brazil

**Keywords:** bromocriptine, cabergoline, dopamine, hyperprolactinemia, prolactinoma, resistance, treatment

## Abstract

**Introduction:** Prolactinomas are preferentially treated with dopamine agonists. However, a few adenomas are resistant to this treatment.

**Objective:** To evaluate the characteristics of patients with resistance to dopamine agonists in the long-term.

**Method:** A retrospective study of six cases was made. Patients who did not achieve normalized prolactin blood concentrations and a reduction of more than 50% of the tumor volume with the minimum dose of 3.5 mg per week of cabergoline for 3 months or the maximum supported dose of bromocriptine for 6 months were considered resistant to dopamine agonists. Patients were followed up at the Clinic of Neurology and Endocrinology or the University Hospital of Brasilia.

**Results:** Six patients were selected. Three patients were initially treated with bromocriptine prior to treatment with cabergoline. Four patients were men, and two were women. At the time of diagnosis, ages ranged from 9 to 62 years. Initial prolactin concentrations ranged from 430 to 14,992 ng/mL and in the last assessment ranged from 29.6 to 2,169 ng/mL. The tumor volume ranged from 0.77 to 24.0 mm^3^. Tumor regression occurred in all patients, ranging from 20 to 100%, but total disappearance of the adenoma with an empty sella occurred in one patient. The maximum weekly doses of cabergoline ranged from 3.0 to 4.5 mg. Follow-up time ranged from seven to 17 years. Normalization of prolactin concentrations occurred only in one woman after 17 years of treatment. Three patients also underwent surgery, but only one woman was cured of the disease.

**Conclusion:** This study confirms that tumors resistant to dopamine agonists are more aggressive, since we did not have any microadenoma; treatment with high dose of cabergoline may reduce the size of the tumor without its disappearance, and that normalization of prolactin concentration rarely occurs. To our knowledge, this is the longest follow-up of a series of cases with resistance to dopamine agonists.

## Introduction

Prolactinomas are pituitary tumors that originate from lactotroph cells. Due to the excessive production of prolactin, they mainly cause menstrual alterations and galactorrhea in women and decreased libido in men (1, 2). They can be classified as microadenomas when smaller than 1 cm and macroadenomas when is 1 cm or more (1, 2). Less than 4% of prolactinomas are giant, that is, with a diameter equal to or greater than 4 cm; these usually compress the optic nerve and invade the sphenoid sinus (2, 3). In general, prolactin levels are related to tumor size: microprolactinomas have lower prolactin concentrations than macroprolactinomas (2).

Treatment of prolactinomas is usually performed with dopamine agonists. Bromocriptine and cabergoline are effective to control prolactin concentrations and reduce the size of the tumor in most patients and are considered more effective than tumor surgery (4–6). According to a review by Oh and Aghi (7), dopamine agonists reduce the size of prolactinomas and the concentration of prolactin through binding to the dopamine D2 receptor in the tumor cells. This causes inhibition of cyclic adenosine monophosphate (cAMP) production due to coupling to the Gαi inhibitory protein leading to a decrease in the size of the cells as a result of the reduction of nuclear, nucleolar, and cytoplasmic areas with involutions of the endoplasmic reticulum and Golgi complex. Furthermore, it leads to cell death (7).

However, some prolactinomas are resistant to treatment with dopamine agonists (7–13). There are many controversies regarding the definition of resistance to dopamine agonists, and this leads to uncertainties when analyzing publications on the subject. One of the reasons for the discrepancies is the variable impact promoted by dopaminergic agonists regarding the reduction of prolactin concentrations and tumor volume. In some cases, treatment of prolactinomas may normalize prolactin concentrations without altering tumor volume or, in rare circumstances, may decrease tumor volume without normalizing prolactin concentrations. Another possibility is to normalize prolactin and shrink the tumor, but signs and symptoms persist, such as galactorrhea or menstrual changes (7–14).

Bromocriptine was the first dopamine agonist used in the treatment of prolactinomas. Resistance to bromocriptine is classically defined by the absence of normalized prolactin concentrations despite the use of a dose equal to or greater than 15 mg per day (15, 16) or 30 mg per day for at least 3 consecutive months (15). Since these high doses of bromocriptine generally cause many side effects, it was determined that resistance to bromocriptine would be considered when there is no normalization of prolactin concentrations after 6 months of treatment with the highest dose tolerated (17, 18).

Regarding cabergoline, resistance has been arbitrarily defined when there is no normalization of prolactin concentration and a reduction in tumor size of <50% at the maximum dose of 2.0 mg per week for 3 months, which is the dose recommended by the manufacturer as the most effective (8–10). However, it has been shown that in many studies there is no quantification of the reduction of tumor size or a wide range of percentages are presented to conclude that treatment with the agonist was successful, e.g., 80, 75, 50, 25, 10%, or no change in tumor size (10). Other authors agree with this definition, but with a dose of cabergoline of up to 3.5 mg per week for 3 consecutive months (7). Some authors have considered that tumor volume should be reduced by at least 30% to be considered significant (11).

Prolactinoma resistance is apparently associated with decreased numbers of dopamine D2 receptors in the cell membrane, but without altering their affinity for dopamine. In addition, there is a decrease in the levels of the G protein which binds the D2 receptor to decrease adenyl cyclase levels which thus further decreases the ability of dopamine to inhibit prolactin secretion [review, (8)]. Furthermore, it has been suggested that the proportion of short isoforms of the D2 receptor, compared to the proportion of the longer isoform, is lower in tumors resistant to dopamine agonists (19). Experimentally, it has been shown that the balance of the phosphatidylinositol 3-kinase (PI3K) and extracellular signal-regulated kinase (ERK) signaling pathways are compromised in prolactinomas and that this may contribute to dopamine resistance (20).

The objective of this study was to describe six cases of dopamine agonist resistance with long-term follow-up, from seven to 17 years, in which the clinical and laboratory characteristics and the results of the long-term treatment of these patients were analyzed.

## Methods

Six patients with a diagnosis of prolactinoma resistant to dopamine agonists were retrospectively evaluated for periods ranging from seven to 17 years. Five patients were followed at the Clinic of Neurology and Endocrinology and one at the University Hospital of Brasilia, in Brasilia, DF.

Patients who did not achieve normalized prolactin blood concentrations and a reduction in tumor volume above 50% at a dose of 3.5 mg or more per week were considered resistant to cabergoline (7). Resistance to bromocriptine was considered when there was no normalization of prolactin concentration after 6 months of use at the maximum dose tolerated by the patient (17, 18).

When questioned, all patients referred to the regular use of a dopaminergic agonist.

Hormonal and metabolic assessments were performed on the blood collected from the antecubital vein after nocturnal fasting for 10 to 12 h. Prolactin concentrations were determined using a chemiluminometric immunoassay/IMMULITE 2000 (normal values = 2.5 to 17 ng/mL for men and 1.9 to 25 ng/mL for women); macroprolactin was investigated using the polyethylene glycol precipitation method and it was considered not present when a recovery of more than 65% occurred. Chemiluminescence was used to measure the following parameters: follicle stimulating hormone (FSH; normal values = 1.0 to 8.0 mIU/mL for men and 1.0 to 8.0 mIU/mL for women in the follicular phase of the cycle), luteinizing hormone (LH; normal values = 2.0 to 12.0 mIU/mL for men and 2.0 to 12.0 mIU/mL for women in the follicular phase of the cycle), estradiol (normal values = follicular phase 9 to 221 pg/mL, middle of the cycle 83 to 689 pg/mL, and luteal phase 26 to 408 pg/mL), total testosterone (normal values = 212 to 755 ng/dL), thyroid stimulating hormone (TSH; normal values = 0.35 to 5.5 μUI/mL), free thyroxine (FT4; normal values = 0.7 to 1.8 ng/dL), and cortisol (normal values at 8 AM = 6 to 25 μg/dL).

Diagnoses of hormonal deficiencies were made using clinical and laboratory assessments as follows: for basal cortisol, values lower than 6 μg/dL or the use of exogenous corticosteroids for hormone replacement were considered as suggestive of adrenal insufficiency; levels higher than 10 μg/dL were considered as normal, and in cases of basal cortisol between 6 and 10 μg/dL or clinically suspicious of adrenal insufficiency, were submitted to a insulin tolerance test, considered as normal, if levels > 18 μg/dL were achieved (21); for thyroid function, FT4 values lower than 1.0 ng/mL when hypothyroidism secondary to the hypothalamic-pituitary lesion was suspected (22), TSH above 10 μUI/mL in cases of primary thyroid insufficiency, or if the patient was on levothyroxine replacement therapy; for testicular deficiency, a total testosterone concentration below 300 ng/dL on two occasions, decreased libido, and erectile dysfunction symptoms or on testosterone replacement (23); and for ovarian deficiency, estradiol values below the reference values for the menstrual cycle or for menopausal women.

Regarding the classification of the tumors by magnetic resonance imaging (MRI), the classification of Edal et al. was used (24). In the upper extension: degree 0—tumor without extension to suprasellar cistern; grade 1—tumor extension to suprasellar cistern, without contact with optic chiasm; grade 2—tumor in contact with optic chiasm, without displacement; grade 3—tumor displacing the optic chiasm; grade 4—obstructive hydrocephalus. In the lower extension: degree 0—sellar floor intact; grade 1—inferior focal dilatation of the sellar cavity as an indirect sign of perforation of the sellar floor; grade 2—tumor penetration into the sphenoid sinus. In the previous extension: degree 0—tumor does not extend perpendicular line tangent to the tubercle sellar grade 1—tumor exceeds perpendicular line tangent to the tubercle seal with extension to anterior cranial fossa. In the posterior extension: degree 0—tumor without extension for infero-posterior region to the back of the sellar cavity; grade 1—tumor with extension to infero-posterior region to the back of the sellar or clivus. In the parasselar extension: degree 0: tumor without involvement of cavernous sinuses; grade 1: tumor does not extend beyond the tangent line passing through the centers of the two segments of the internal carotid artery; grade 2: tumor does not extend beyond the tangent line to the lateral margins of the segments of the internal carotid artery; grade 3: tumor extends beyond the tangent line to the lateral margins of the segments of the internal carotid artery; grade 4: tumor totally encompasses intra-cavernous carotid artery. Degrees 3 and 4 most likely demonstrate invasion of cavernous sinuses. Tumor volume was calculated using the formula: (cranio-caudal diameters x laterolateral x antero-posterior) / 2.

This study was approved by the Ethics Committee of the Verhum Clinic, Brasilia-DF. The patients provided written informed consent for the publication of this article.

## Description of the cases

As shown in Table 1, there were four male and two female patients. At the time of diagnosis, ages ranged from 9 to 62 years. Initial prolactin concentrations ranged from 430 to 14,992 ng/mL and at the end of the last assessment ranged from 29.6 to 2,169 ng/mL. Only patient 6 achieved a normalized prolactin concentration after 17 years of treatment with a dopamine agonist. Case 5 only achieved a normalized prolactin concentration (4.9 ng/mL) after surgical removal of the tumor. Three patients had a giant tumor, i.e., larger than 4 cm, and three had macroadenomas. The tumor volume ranged from 0.77 to 24.0 mm^3^. Tumor regression occurred in all patients, ranging from 20 to 100%, but total disappearance of the adenoma with an empty sella occurred in one patient. The patient 6 was the one who normalized prolactin and the tumor disappeared after 17 years of treatment. All patients were treated with cabergoline, but three of them received bromocriptine as first treatment (cases 3, 4, and 6). Initial doses of cabergoline ranged from 1.0 to 2.0 mg per week, and maximum doses ranged from 3.0 to 4.5 mg per week. Three patients underwent surgery: case 3 had an initial transcranial approach with biopsy of the tumor, followed by treatment with bromocriptine and then cabergoline; case 4 started treatment with bromocriptine which was replaced by cabergoline and subsequently underwent transsphenoidal surgery and radiotherapy; and case 5 was treated with cabergoline, and the tumor was removed by transsphenoidal surgery. Assessment of macroprolactin was performed on all patients on several occasions, but it was always negative.

**Table 1:** Clinical and laboratory characteristics of the six patients with resistance to dopamine agonists.

**Cases**	**1**	**2**	3^*^^†^	4^*^^†^	**5^†^**	6^*^
Gender	M	M	M	M	F	F
Age (years)	54	62	9	50	22	22
**Prolactin (ng/mL)**						
Initial	1,947	14,992	2,400	2,600	659	430
Final	353	2,169	44.5	765	367	29.6
**Tumor (mm**^3^**)**						
Initial	3.75	12.5	24.0	giant^‡^	0.77	12.6
Final (%)^§^	95.0	90.0	90.0	90.0	20.0	100.0
**Cabergoline (mg/week)**						
Initial	1.5	1.0	1.0	1.5	2.0	1.0
Maximum	3.5	3.5	4.5	3.5	3.5	3.0
Final	2.0	2.0	4.5	3.5	–	1.5
Time (months)	180	162	179	180	88	201

* patients initially used bromocriptine;

† underwent surgery;

‡ measures not available;

§ *percentage reduction of tumor*.

### Case 1

A 54-year-old white male patient presented with decreased libido and slow thinking that started 6 years before and worsened over the last 4 years. He did not report visual complaints or headaches. He had a height of 1.70 m, a weight of 68 kg, and a BMI of 23.5 kg/m^2^. He presented with paleness of the skin and mucous membranes, dry skin, and weak and brittle nails. He had a blood pressure of 90/60 mmHg. Campimetry was normal.

**Figure 1:**
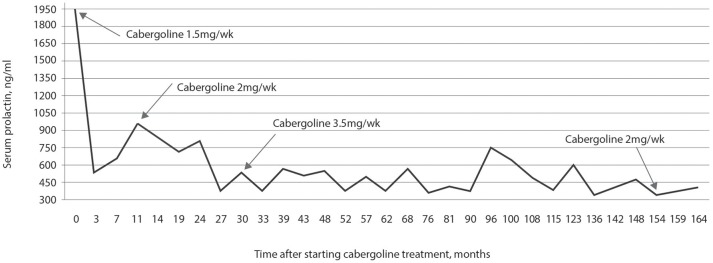
Prolactin concentration in response to treatment with cabergoline over 14 years and 6 months. Prolactin concentrations were never normalized, even while using a dose of 3.5 mg per week of cabergoline for 10 years; after 13 years of treatment, decreasing the weekly dose to 2 mg maintained prolactin concentrations at values similar to those observed for a dose of 3.5 mg.

Initial hormonal assessments showed a prolactin level of 1,947 ng/mL, LH of 1.6 mIU/mL, FSH of 1.3 mIU/mL, and total testosterone of 258 ng/dL. As shown in Figure 1, the use of weekly cabergoline doses of 1.5 mg resulted in a nearly two-thirds decrease in prolactin concentration in the first 3 months. However, in subsequent follow-ups, weekly doses of 2.0 mg and up to 3.5 mg did not normalize the prolactin concentration. Over the last 10 months, there was no change in prolactin concentrations when the reduced weekly dose of 2.0 mg was compared to the 3.5 mg dose that was used for 124 months.

After 4 months of treatment with cabergoline, the concentration of testosterone was 186 ng/dL, and intramuscular replacement therapy with testosterone propionate, testosterone fempropionate, testosterone isocaproate, and testosterone decanoate was performed every 21 days; the patient reported improvement of sexual dysfunction. After starting the testosterone replacement therapy, testosterone concentrations ranged from 471 to 598 ng/dL. PSA was always below 2.53 ng/mL.

The initial concentration of cortisol was 1 μg/dL, and replacement therapy was started with 5 mg of prednisone. After 1 year, recovery was observed with cortisol concentrations of 13 μg/dL and adrenocorticotropic hormone (ACTH) of 28.4 pg/mL, and the replacement therapy was suspended. TSH was 3.62 mIU/mL, FT4 was 1 ng/dL, and treatment with 75 μg levothyroxine was started in the morning. At the last assessment, he used 100 μg levothyroxine and the FT4 was 1.42 ng/dL. Insulin-like growth factor-1 (IGF-1) concentrations were always within the normal limits for the age group of the patient.

The first Doppler echocardiogram was performed after 44 weeks of treatment with cabergoline and showed minimal reflux in the mitral and tricuspid valves. The last assessment, after 120 months, did not show alteration of these valves compared to the first assessment.

Since the start of the treatment with cabergoline and the hormone replacement therapy, the patient's thinking improved, his dry and pale skin improved, and he became more active, including running on the street daily.

**Figure 2:**
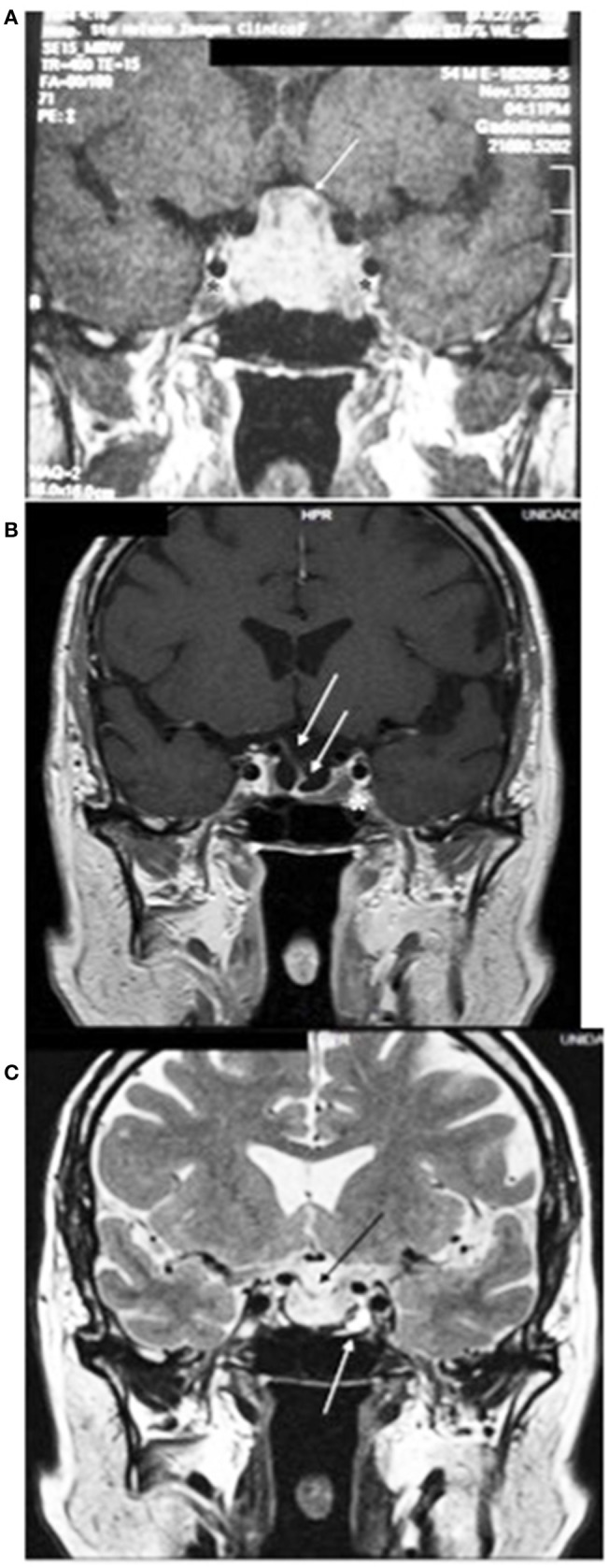
**(A)** Magnetic resonance imaging of the sella turcica before starting the treatment with cabergoline, on T1 coronal, with the tumor measuring 25 × 20 × 15 mm, with heterogeneous contrast, with cystic appearance, bilaterally invading the cavernous sinuses (^*^) and compressing the optic chiasm (white line). **(B)** After 14 years of treatment, with heterogeneous impregnation of the contrast medium, a small lesion is visible with a close association with the left carotid (^*^), invagination of the optic chiasm, and the pituitary stalk deviated to the right (white arrows). **(C)** T2 image shows a small lesion, with a hypersignal (white arrow), with cystic appearance, and invagination of the optic chiasm (black arrow).

MRI of the sella turcica showed a pituitary macroadenoma with invasion of the cavernous sinuses and compression of the optic chiasm, as shown in Figure 2A. Magnetic resonance images of the sella turcica, obtained after 14 years of treatment, are presented in Figures 2B,C and show a small lesion with cystic appearance near the left carotid.

### Case 2

**Figure 3:**
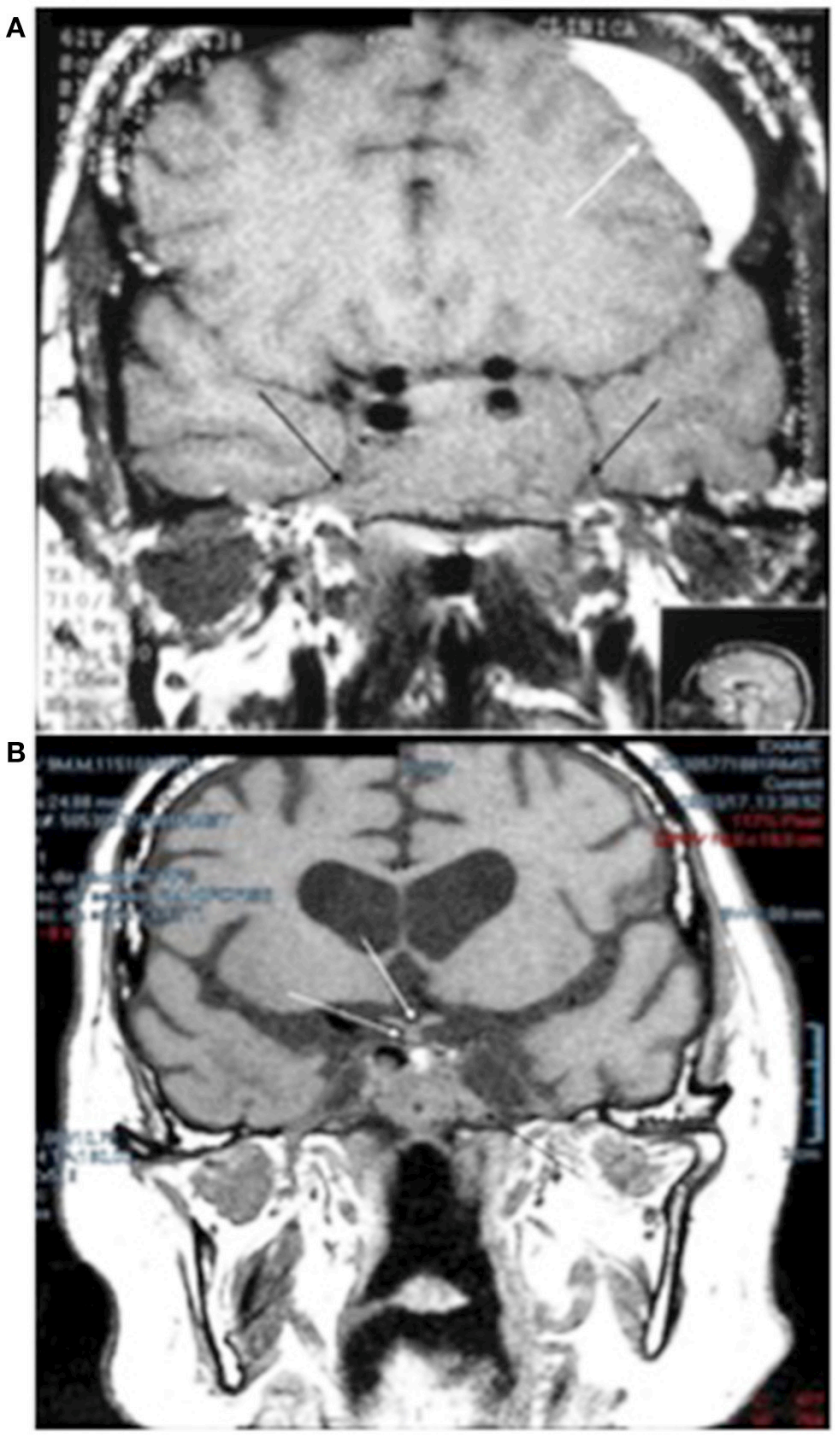
**(A)** Magnetic resonance imaging of the sella turcica, on T1, without contrast, prior to starting treatment with cabergoline, which shows a pituitary adenoma with total invasion of the sphenoid sinus and cavernous sinuses on both sides (black arrows), measuring 4.0 × 2.5 x 2.5 cm. The image also shows a subacute subdural hematoma in the left frontal lobe of the brain (white arrow). **(B)** After 17 years of treatment with cabergoline, a reduction in the size of the pituitary adenoma can be seen with visualization of the optic chiasm and pituitary stalk (white arrows); tumor remnants invading the sphenoid sinus (black arrow) are visible.

A 62-year-old white male patient presented with type 2 diabetes mellitus, dyslipidemia, multiple valvopathies, systemic arterial hypertension, and subacute subdural hematoma of the left frontal lobe. During preoperative examinations for subclinical subdural hematoma drainage, MRI was performed which confirmed a large solid tumor lesion in the hypothalamic-pituitary region, as described in Figure 3A.

In the systematic interrogation, he mentioned decreased libido and sexual impotence for the past 14 years. He had no visual complaints, was practicing target shooting, and campimetry was normal. He had a height of 1.67 m, a weight of 71.5 kg, and a BMI of 25 kg/m^2^. He had a pancardiac systolic murmur +++/4+. Blood pressure was 130/90 mmHg. Doppler echocardiography showed a double aortic valve injury with predominance of stenosis, concentric left ventricular hypertrophy with normal global and segmental systolic function, and left ventricular diastolic dysfunction.

**Figure 4:**
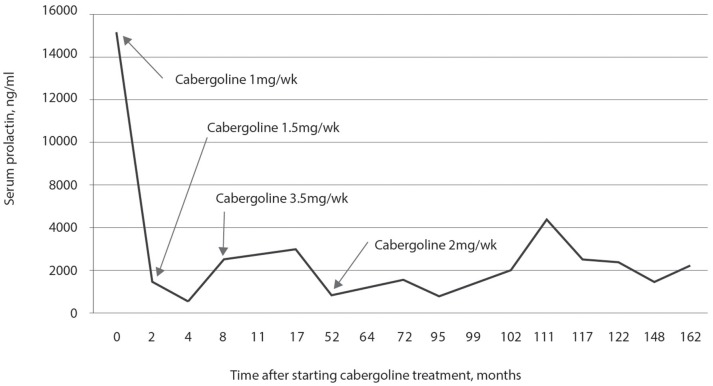
Prolactin concentration in response to treatment with cabergoline for 14 years. A significant decrease in the concentration of prolactin was observed with a dose of 1.0 mg per week of cabergoline in the first 2 months, but without normalization of prolactin concentration. Prolactin concentration was not normalized with a dose of 3.5 mg per week used for 44 weeks, and it remained elevated until the last assessment; after 102 weeks, treatment with quetiapine and mirtazapine was started, which caused an increase in the prolactin concentration.

Initial hormonal assessment showed a prolactin concentration of 14,992 ng/mL, FSH of 1.84 mIU/mL, LH of 1.2 mIU/mL, and total testosterone of 260 ng/dL. Treatment with cabergoline was initiated, and the assessment of prolactin concentration is presented in Figure 4. Treatment started with 1.0 mg per week, and there was a substantial reduction in the concentration of prolactin after 2 months: from 14,992 to 1,712 ng/mL. However, subsequent treatment was not sufficient to decrease prolactin concentrations as strongly; despite increasing the dose to 3.5 mg per week for 48 months, prolactin levels remained at 840 ng/mL. From 52 weeks of treatment to the last assessment at 162 weeks, he used 2.0 mg per week of cabergoline and his prolactin concentration remained above 1,000 ng/mL. However, at 70 years of age and after 95 months of treatment with cabergoline, he had a cerebrovascular accident due to an aneurysm rupture. One year after this event, he developed psychiatric changes that were controlled with quetiapine and mirtazapine. Increased prolactin concentrations between weeks 102 and 148 were most likely due to the concomitant use of these two drugs in addition to 2 mg of cabergoline.

After 4 months of treatment, the patient still complained of sexual impotence, and intramuscular replacement therapy with testosterone propionate, testosterone fempropionate, testosterone isocaproate, and testosterone decanoate was performed every 21 days. The patient reported improvements regarding sexual dysfunction. In the last two years, he underwent replacement therapy using a 2% testosterone gel applied to both armpits. After starting the hormone replacement therapy, testosterone levels increased from 480 to 670 ng/dL, FSH from <0.07 to 3.78 mIU/mL, and LH from <0.07 to 3.49 mIU/mL.

Assessment of thyroid function showed TSH ranging from 1.54 to 1.86 mIU/mL and FT4 from 0.94 to 1.1 ng/dL. Hypothyroidism secondary to the hypothalamic and pituitary lesion with TSH of 1.7 μIU/mL and FT4 of 0.83 ng/dL was observed 2 years later, and replacement therapy with 50 μg of levothyroxine was started. Assessment of adrenal function showed cortisol levels at 8 h ranging from 16.3 to 25 μg/dL. IGF-1 concentration was always normal for the age group.

In addition to cabergoline, quetiapine, and mirtazapine, the following drugs were used together: hydrochlorothiazide, losartan, nifedipine, bisoprolol, indapamide, potassium chloride, aspirin, and rosuvastatin.

MRI performed after 17 years of treatment is shown in Figure 4A and shows the almost complete disappearance of the tumor (Figure 4B).

### Case 3

**Figure 5:**
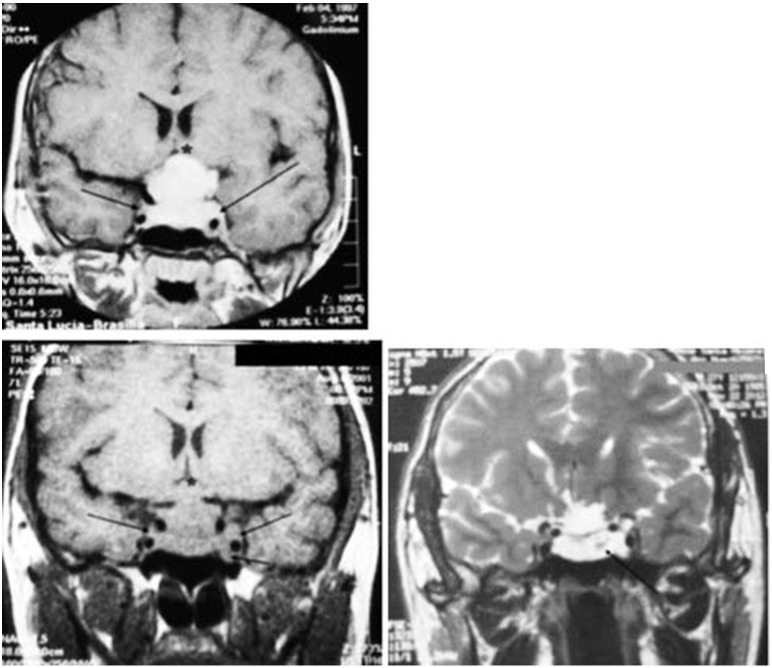
Magnetic resonance imaging of the sella turcica on T1 coronal, with homogeneous contrast, performed before transcranial surgery and the start of the treatment with bromocriptine, which shows the pituitary adenoma with invasion of the cavernous sinus on both sides (black arrows) and compression of the optic chiasm (^*^). Without contrast, performed 41 months after starting the treatment with dopamine agonists (34 months of using bromocriptine and 7 months of using cabergoline), showing the pituitary adenoma with invasion of the cavernous sinus on both sides (black arrows) and compression of the optic chiasm. On T2, 14 years after starting the treatment with dopamine agonists, there is a marked decrease in the size of the pituitary adenoma with formation of a cystic mass (black arrow).

A 9-year-old white male patient underwent MRI during investigations for short stature. Findings showed a tumor mass in the hypothalamic-pituitary region, as described in Figure 5A. He underwent transcranial surgery, and a biopsy of the lesion was performed. Histochemical analysis showed that it was a pituitary tumor and the concentration of prolactin was 2,400 ng/mL.

**Figure 6:**
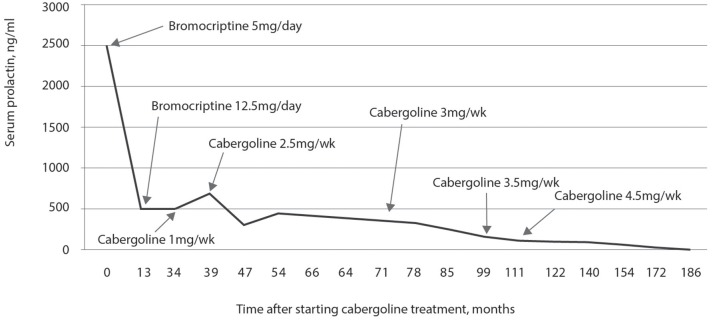
Plasma prolactin concentrations during 15 years of treatment with dopaminergic agonists. During the first 34 months there was a significant reduction with the use of bromocriptine (final dose of 12.5 mg per day). In the 34th month, treatment with 2 mg per week of cabergoline was started and there was a significant reduction in prolactin after only 20 months of treatment with 2.5 mg per week of cabergoline. The reduction was most significant when the dose of cabergoline was increased to 4.5 mg per week after 111 months of treatment with dopaminergic agonists.

Treatment started with bromocriptine, 5.0 mg per day, and he was later treated with cabergoline. The assessments of prolactin concentration in response to treatment with bromocriptine is shown in Figure 6. After 13 months of treatment, the concentration of prolactin was 360 ng/mL, and the dose of bromocriptine was increased to 12.5 mg per day. After 34 months of treatment with bromocriptine, the concentration of prolactin was 493 ng/mL.

He was then diagnosed as having resistance to bromocriptine, and the medication was replaced by 1.0 mg per week of cabergoline. After 5 months, the concentration of prolactin was 668 ng/mL, and the dose of cabergoline was increased to 2.5 mg per week. During the follow-up, the dose of cabergoline was gradually increased and the maximum dose used was 4.5 mg per week from month 115 of treatment. After 145 months of treatment, the concentration of prolactin was 44.57 ng/mL. The patient completed 15 years of treatment including the period he was using bromocriptine.

At age 14, he had a height of 1.52 m, a weight of 48 kg, and was in stage III of Tanner. The bone age was 15 years. At age 15, he had a height of 1.54 m, showing that the growth rate remained low for the age group. Development of secondary sexual characteristics occurred without hormonal intervention. At that time, the concentration of IGF-1 was 170 ng/mL, which is considered low for the age group; FSH concentration was 2.3 mIU/mL, LH was 2.1 mIU/mL, testosterone was 470 ng/dL, TSH was 1.6 mIU/mL, FT4 was 1.3 ng/dL, and cortisol was 11.3 μg/dL. Assessments of GH secretion using stimulation tests of insulin- and clonidine-induced hypoglycemia showed deficiencies in secretion. GH replacement therapy was started subcutaneously at a dose of 5.0 IU per day. At age 16, using GH, the concentration of IGF-1 was 600 ng/mL, normal for the age group. The patient reached a final height of 1.62 m. At the last assessment, at age 28, the concentration of IGF-1 was 255.6 ng/mL, a result considered normal for the age group of the patient. Assessment of testicular function showed testosterone concentrations ranging from 241 to 670 ng/dL, FSH from 1.84 to 3.78 mIU/mL, and LH from 1.2 to 3.49 mIU/mL. Assessment of thyroid function was always normal with TSH ranging from 1.1 to 3.1 mIU/mL and FT4 from 0.9 to 1.3 ng/dL. Assessment of adrenal function was always normal, with cortisol levels ranging from 9.7 to 20.9 μg/dL and ACTH ranging from 25 to 55 pg/mL. During the insulin-induced hypoglycemia test, a cortisol concentration of 30 μg/dL was observed.

The series of MRI of the sella turcica taken 41 months after treatment shows that the tumor remained virtually unchanged (Figure 5B). After 14 years, significant cystic degeneration of the tumor can be observed (Figure 5C).

### Case 4

A 50-year-old brown male patient complained of left ear problems, with otalgia, clogged ear, and tinnitus. He also had bloody nasal discharge and sputum. These symptoms started occurring 1 year before. His libido had decreased 5 years earlier. He did not have headaches. During the investigation, MRI of the sella turcica region was performed and showed a huge tumor mass with invasion of the sphenoid sinuses and nasal cavity, without suprasellar extension. There was also osteomastoiditis on the left. Sarcoidosis or Wegener's granulomatosis was initially suspected (images not shown).

**Figure 7:**
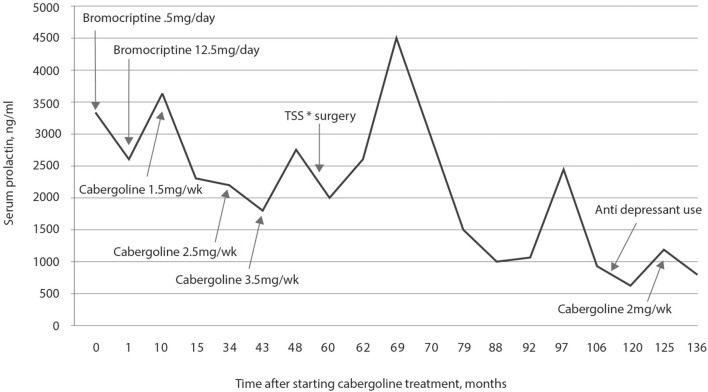
Plasma prolactin concentrations during 15 years of treatment with dopamine agonists. During the 5 months of treatment with bromocriptine, there was a decrease in prolactin concentration but without normalization; with cabergoline, even at doses of 3.5 mg per week, prolactin normalization did not occur. After transsphenoidal surgery, there was a decrease in prolactin concentration, but without normalization; decreasing the dose to 2.0 mg per week in the last year of follow-up resulted in a slight increase in prolactin concentrations.

The initial hormonal assessment showed a prolactin concentration of 2,600 ng/mL, LH of 1.31 mIU/mL, FSH of 2.03 mIU/mL, and total testosterone of 416 ng/dL. Changes in prolactin concentration during the 15 years of treatment with dopamine agonists are shown in Figure 7. A dose of 12.5 mg of bromocriptine was associated with a marked decrease in prolactin concentration (691 ng/mL) after 5 months of treatment, yet without normalizing it. It was then replaced by cabergoline at a dose of 1.5 mg per week for 3 years, with the maintenance of a high prolactin concentration up to 3,600 ng/mL. Increasing the dose to 2.5 mg for 2 months and then to 3.5 mg per week for 10 years failed to normalize the prolactin concentration. Six years after starting the treatment, the concentration of prolactin was 4,470 ng/mL, and the patient underwent transsphenoidal surgery and radiotherapy. After these events, prolactin concentration decreased but was not normalized. Decreasing the dose to 2.5 mg per week during the last 12 months of observation resulted in a slight increase in prolactin concentration.

With 1.5 mg of cabergoline per week, there was a progressive improvement in tinnitus and hearing loss; however, these symptoms persisted. After 2 years, retroauricular and left ear pains became very intense; ethmoid, maxillary, and frontal sinus inflammation was observed, which improved with clarithromycin treatment.

Eighteen months after starting the treatment, testosterone levels were low, and he continued to complain of decreased libido; replacement therapy with testosterone propionate, testosterone fempropionate, testosterone isocaproate, and testosterone decanoate was started. This replacement therapy was maintained until the last assessment, after 15 years of treatment. The PSA always remained normal.

At the first assessment, he had a TSH concentration of 18.14 mIU/mL, FT4 of 0.77 ng/dL, and anti-thyroperoxidase antibody of 1,715 IU/mL. Replacement therapy with 75 μg of levothyroxine was started when fasting, which continued until the last assessment, after 15 years of treatment.

Before the transsphenoidal surgery, 6 years after starting the treatment, secretions of cortisol and ACTH were normal and, despite having low blood pressure (90/60 mmHg), there were no signs or symptoms of adrenal deficiencies. After surgery, replacement therapy with 5 mg of prednisone in the morning was started, which was discontinued after 1 year with no signs or symptoms of hormone deficiencies. Eight years later, it was necessary to resume the replacement therapy with 3 mg of prednisolone due to severe asthenia and postural hypotension. The initial concentration of IGF-1 was 187 ng/mL, and it ranged from 115 to 221 ng/mL, considered adequate for the age group.

Fourteen years after starting the treatment with dopamine agonists, including 10 years of using 3.5 mg of cabergoline, a Doppler echocardiogram showed a tricuspid valve with mild reflux.

Assessment of bone mineral density after 2 and 9 years of follow-up was compatible with lumbar spinal osteoporosis. Therapy with risedronate was started.

The patient developed severe depression symptoms after 4 years of treatment with cabergoline. He used 10 mg of escitalopram oxalate and 1 mg of alprazolam for 4 years. Escitalopram was replaced with 25 mg of agomelatine for 2 years. In the last five years, he used 100 mg of pregabalin twice a day and 1 mg flunitrazepam.

Assessment of tumor images before the treatment show an enormous tumor with invasion of the sphenoid sinus and nasal cavity; however, there was no suprasellar growth, and the optic chiasm was always preserved. After using 12.5 mg of bromocriptine for 5 months and 1.5 mg of cabergoline for 20 months, the images still show the sphenoid sinus occupied with heterogeneous material; nevertheless, a reduction in the tumor size was already evident because the pituitary was already visible and occupied the ventral part of the sella cavity, and it was possible to identify the anterior and posterior pituitary and the pituitary stalk (images not shown).

**Figure 8:**
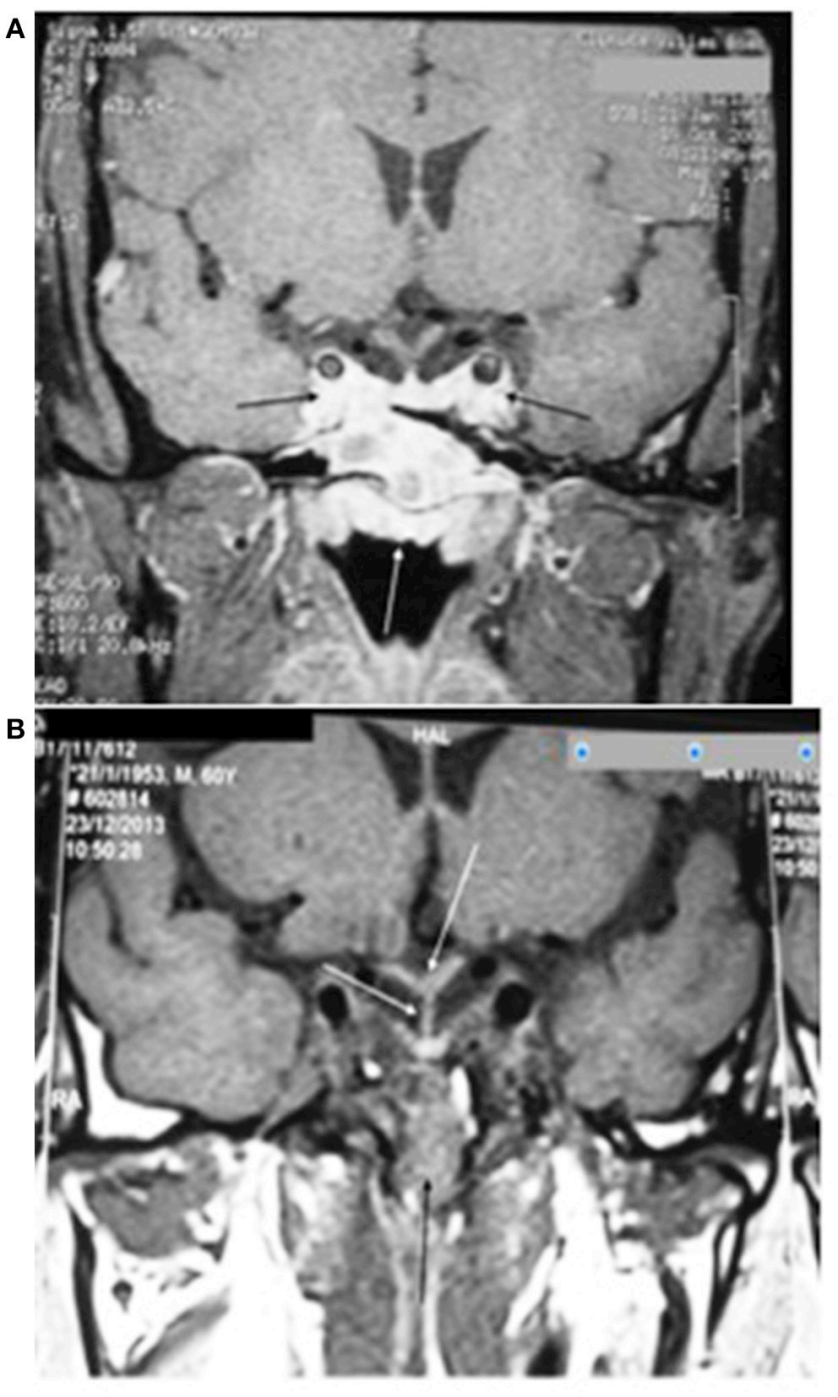
**(A)** Magnetic resonance imaging of the sella turcica on T1 coronal with heterogeneous contrast after 52 months of treatment with dopamine agonists shows a reduction in the pituitary adenoma with invasion of the cavernous sinuses (black arrows) and total invasion of the sphenoid sinus (white arrow). **(B)** After 11 years of treatment with dopamine agonists and 5 years after transsphenoidal resection and radiotherapy, the pituitary adenoma is almost totally reduced, but still present in the sphenoid sinus (black arrow); the centered pituitary stalk and pituitary gland and invagination of the optic chiasm (white arrows) can also be seen.

MRI performed 52 months after starting the treatment with dopamine agonists is shown in Figure 8A. The lesion was reduced compared to the initial lesion, the pituitary and pituitary stalk appeared normal, the expansive lesion accompanied the temporal and occipital bones throughout the clivus and the sphenoid plane, the mass had inferior and anterior components in the rhinopharynx, and there were no signs of carotid envelopment.

The patient underwent surgery through the transsphenoidal route, 7 years after starting the treatment, and received radiotherapy with 4,500 rads after 8 years of treatment. Images obtained 1 month after the surgery still show a lesion in the clivus, extending to the sphenoid sinus and the rhinopharynx (images not shown).

Eleven years after starting the treatment with dopamine agonists and 5 years after surgery and radiotherapy, the tumor almost disappeared, although there were still alterations in the sphenoid sinus and heterogeneous contrast in the clivus. Figure 8B illustrates this situation with the remaining tumor in the sphenoid sinus but showing the pituitary and optic chiasm.

### Case 5

**Figure 9:**
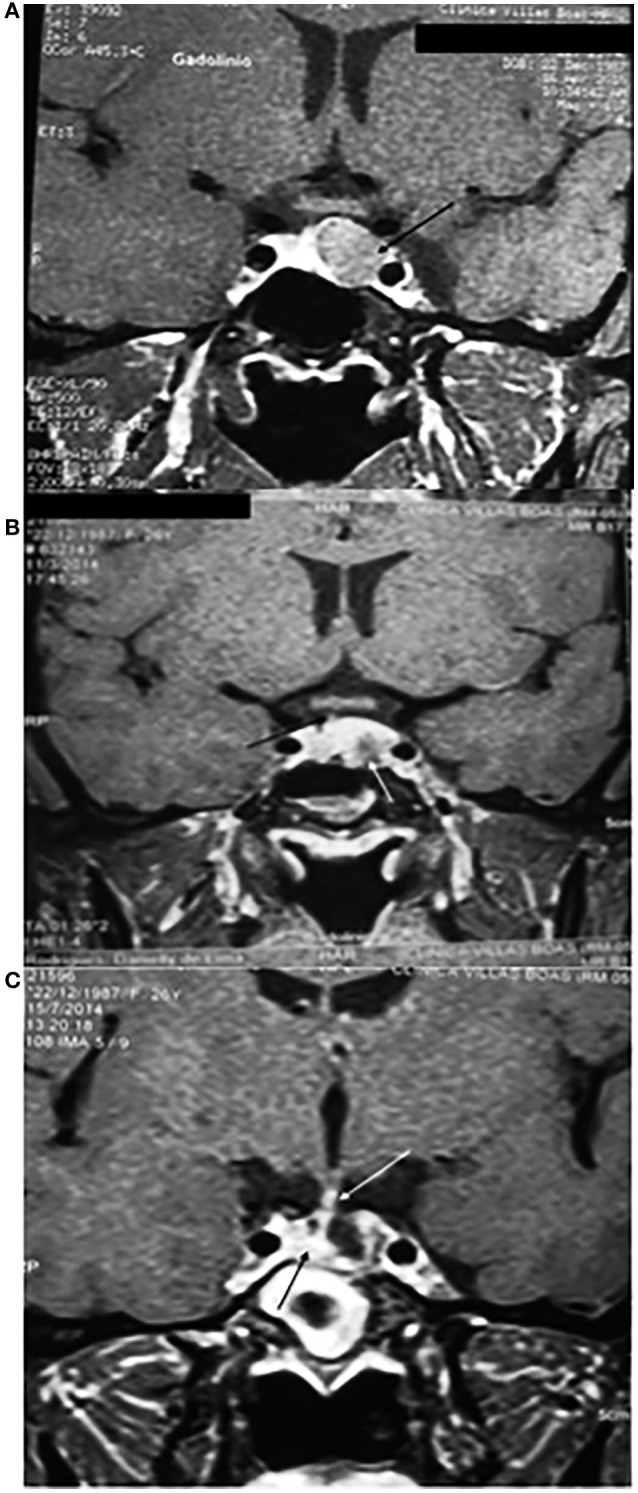
**(A)** Magnetic resonance imaging of the sella turcica on T1 coronal with contrast before treatment with cabergoline showing a lesion with heterogeneous contrast measuring 1.0 × 1.4 × 1.1 cm, without invasion of the cavernous sinus, but with a close association with the left carotid (black arrow). **(B)** Imaging performed 47 months after starting the treatment with 2.0 mg per week of cabergoline and 2 months before the transsphenoidal surgery showing the pituitary adenoma with a close association with the left carotid artery (white arrow) and the pituitary stalk diverted to the right (black arrow). **(C)** Three months after the transsphenoidal surgery, the pituitary adenoma was completely removed with the stalk (white arrow) and the pituitary (black arrow) diverted to the right.

A 22-year-old white female patient reported menarche at age 12, with irregular cycles and menstrual delays of up to 6 months. She had acne that worsened when she was 18 years old. The initial hormonal assessment showed a prolactin concentration of 659 ng/mL. MRI of the sella turcica was performed confirming a pituitary tumor, as shown in Figure 9A.

**Figure 10:**
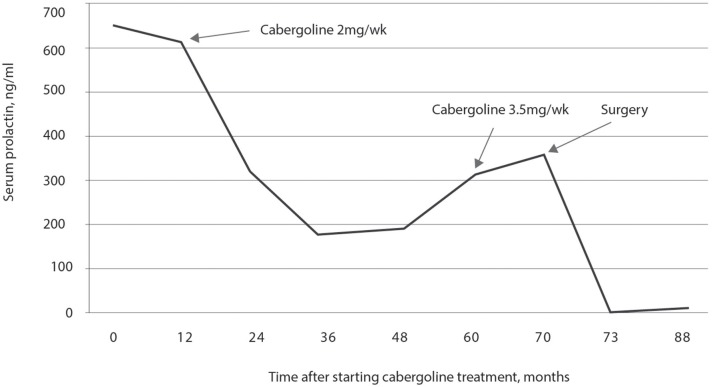
Prolactin concentration in response to treatment with 2.0 mg per week of cabergoline. A progressive decrease in prolactin concentrations was observed up to 36 months of treatment, remaining stable for 12 months, and then gradually increasing to reach the maximum value at 70 months of treatment when transsphenoidal surgery was performed with resolution of hyperprolactinemia.

Treatment was started with 2.0 mg per week of cabergoline. The assessment of prolactin concentration in response to cabergoline is shown in Figure 10. During the follow-up, she still presented menstrual irregularities and galactorrhea, and prolactin concentration remained elevated. Approximately 3 years after starting the treatment, she presented intense headaches that lasted a few hours. After 4 years of treatment, using 2.0 mg per week of cabergoline, the concentration of prolactin was 189 ng/mL. After 5 years of treatment, she was reassessed at the current service and had a height of 1.54 m, a weight of 69 kg, and a BMI of 29.11 kg/m^2^. While using 2.0 mg per week of cabergoline, hormonal measurements showed a prolactin concentration of 311 ng/mL, FSH of 4 mIU/mL, LH of 7.1 mIU/mL, and estradiol of 22.3 pg/mL. The dose of cabergoline was then increased to 3.5 mg per week and, after 10 months of using that dose and 6 years after starting the treatment, the concentration of prolactin was 367 ng/mL. The patient then underwent transsphenoidal surgery resulting in the disappearance of the galactorrhea and normalization of the menstrual cycles. Prolactin concentrations analyzed three and 18 months after the surgical procedure were 1.9 and 4.9 ng/mL, respectively (Figure 10).

Figure 9A shows the MRI of the sella turcica prior to treatment with cabergoline, which shows a lesion with heterogeneous contrast without invasion of the cavernous sinus but with close association with the left carotid artery. Four years after starting the treatment with cabergoline and 2 months before the surgery, there was no regression of the tumor (Figure 9B). Three months after the transsphenoidal surgery, the pituitary adenoma was completely removed, and the stalk and pituitary remained diverted to the right (Figure 9C).

### Case 6

A 22-year-old white female patient complained of dizziness, nausea, and vertigo for the last 4 years; she was diagnosed with labyrinthitis. Treatment with flunarizine was initiated and the condition partially improved, except nausea that progressively worsened. She also presented galactorrhea and amenorrhea for the past 4 years. Menarche occurred at age 15 and menstrual cycles remained irregular for 6 months; since then, she presented with amenorrhea. Regarding her family history, she reported having a fourth cousin with a pituitary tumor (case 3 of the present study). She had a height of 1.59 m, a weight of 60 kg, and a BMI of 23.8 kg/m^2^. She had a blood pressure of 120/70 mmHg, and galactorrhea was observed.

**Figure 11:**
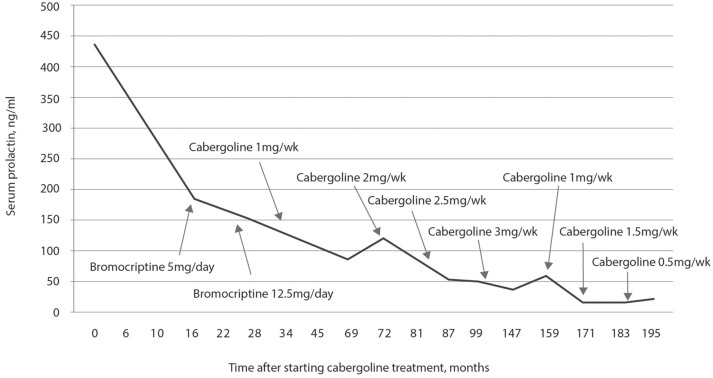
Plasma prolactin concentrations during treatment with dopamine agonists. It was observed that even with a dose of 12.5 mg of bromocriptine over 28 months, prolactin concentrations were not normalized; replacement with cabergoline only normalized prolactin concentrations 14 years after starting the treatment with this drug.

The initial hormonal assessment showed a prolactin concentration of 430 ng/mL, FSH of 4.1 mIU/mL, and LH of 2.2 mIU/mL. Treatment with 2.5 mg bromocriptine per day was started. Prolactin concentration in response to treatment with bromocriptine is shown in Figure 11. The concentration of prolactin 6 months after starting the treatment was 354 ng/mL. The dose of bromocriptine was increased every 6 months at subsequent visits to 5.0, 10, and 12.5 mg per day. However, after 28 months of follow-up, the concentration of prolactin was 155 ng/mL and, therefore, the patient was diagnosed as having resistance to bromocriptine. This was replaced by 1.0 mg per week of cabergoline. After 4 months on 1.0 mg per week of cabergoline, the concentration of prolactin was 126 ng/mL, and she reported a menstrual cycle which lasted 3 days. At the time, surgery was indicated, but the patient preferred to maintain the drug treatment and continued the treatment with 1.0 mg per week of cabergoline. After 15 months of using cabergoline and 43 weeks after starting the treatment, the concentration of prolactin was 103 ng/mL, and the patient reported regular menstrual cycles, improved libido, and the disappearance of galactorrhea. Since then, the dose of cabergoline has been gradually increased to the maximum dose of 3.0 mg per week, 6 years after starting the use of this agonist. The patient had a collateral effect with increasing dose to 3.5 mg per week: nausea and vomiting. The patient continued using 3.0 mg for 4 years, and the concentration of prolactin was 34.17 ng/mL. The dose of cabergoline was reduced to 1.0 mg per week, but the concentration of prolactin increased to 67 ng/dL. Increasing the dose to 1.5 mg for 1 year decreased the concentration of prolactin to 18.7 ng/mL. At that time, the dose was decreased to 0.5 mg per week and the final assessment, after 2 years, showed a prolactin concentration of 24.6 ng/mL. This corresponds to 16 years from the start of the treatment with dopamine agonists and 14 years of using cabergoline.

The first assessment of the sella turcica was performed by computed tomography with contrast. A heterogeneous mass was observed in the pituitary region, predominantly on the left, which widened the sella turcica and destroyed the adjacent bone structures, superficially invading the suprasellar cistern and inferiorly the sphenoid sinus. There was no compression of the optic chiasm. The mean size of the lesion was 2.8 × 3.0 × 3.0 cm. Six months after starting the treatment with 2.5 mg per day of bromocriptine, MRI of the sella turcica showed an expansive lesion of 20 × 15 × 12 mm in the left half of the anterior pituitary with invasion of the cavernous sinus, enveloping the left carotid artery and widening the sella floor on that side. The pituitary stalk was diverted to the right.

After 4 months on 1.0 mg per week of cabergoline, MRI of the sella turcica showed an expansive lesion of 12 × 10 × 8 mm, compromising the left half of the anterior pituitary with lateral expansion to the corresponding cavernous sinus. A discrete deviation of the pituitary stalk to the right was observed, without an existing suprasellar component. At the time, surgery was indicated, but the patient preferred to maintain the drug treatment and continued treatment with 1.0 mg per week of cabergoline. After 28 months of treatment with cabergoline, MRI of the sella turcica showed a nodular lesion of 0.4 cm in the left half of the anterior pituitary. She used 3.0 mg per week of cabergoline until completing 116 months of treatment, and the MRI performed on that occasion showed an increase in the size of the sella turcica, which was filled by cerebrospinal fluid with a small volume of pituitary glandular tissue on the floor, featuring a partially empty sella.

The images described above were not presented.

Assessments of thyroid function showed TSH ranging from 1.46 to 3.89 mIU/mL and FT4 ranging from 0.56 to 0.94 mIU/mL, consistent with the diagnosis of secondary hypothyroidism. The patient uses 50 μg per day of levothyroxine.

## Discussion

We present six cases of patients with resistance to dopamine agonists, with a follow-up from seven to 17 years. To our knowledge, this is the longest follow-up of a series of cases with resistance to dopamine agonists. Even with the prolonged use of dopamine agonists, a reduction in tumor size only occurred after lengthy treatment, and normalization of prolactin concentration occurred in only one patient (case 6). In patient 5, prolactin concentrations were controlled, and the tumor disappeared after transsphenoidal surgery.

Three patients started treatment with bromocriptine (cases 3, 4, and 6) and subsequently used cabergoline. Resistance to bromocriptine can be defined when there is no normalization of prolactin concentration after 6 months of treatment with the highest dose tolerated (17, 18). These patients used bromocriptine at the maximally tolerated dose of 12.5 mg per day. Prolactin concentrations were not normalized, despite significant decreases, and the reduction in tumor size was <50% according to the imaging assessments performed at the initial stages of the treatment.

One recommended approach for treating patients with resistance to dopamine agonists is switching to a more potent drug (8). This was done with these patients as they replaced bromocriptine with cabergoline. It is reported that resistance of prolactinomas to bromocriptine is more frequent than resistance to cabergoline. Cabergoline is effective in normalizing prolactin concentrations in 80% of patients resistant to bromocriptine and causes tumor size reductions in 70% of them (25). In a prospective randomized study of women comparing these two agonists, normal prolactin concentrations were obtained in 65% of patients who used bromocriptine and 93% of cabergoline users (25). Another study also showed that cabergoline has a greater effect than bromocriptine in normalizing prolactin concentration and decreasing tumor size after 24 months of treatment, both in macro- and microprolactinomas (5).

Although these patients subsequently used cabergoline, prolactin concentrations were not normalized, and their tumors did not disappear. Case 3 used the maximum dose of 4.5 mg per week and presented with a significantly decreased prolactin concentration after 145 months of treatment; however, levels were not normalized (44.57 ng/mL). Case 4 showed an increased prolactin concentration when changing the treatment to 1.5 mg per week of cabergoline, and even using 3.5 mg did not result in a normalized prolactin concentration (765 ng/mL). Case 6 used a maximum dose of 3.0 mg per week of cabergoline after using bromocriptine for 2 years, and showed a greater reduction in prolactin concentration after 116 months of treatment. Based on the progression of the laboratory assessments, we can conclude that all tumors were resistant to bromocriptine and later to cabergoline.

Three patients (cases 1, 2, and 5) used cabergoline exclusively. However, none of them achieved normalized prolactin concentrations, even after using 3.5 mg per week for a period during the treatment. The hyperprolactinemia of case 5 only resolved after exeresis of the pituitary adenoma. According to Oh and Aghi (7), resistance to cabergoline should only be defined if treatment fails at doses >3.5 mg per week of cabergoline for at least 3 months. This is because using this dose to treat prolactinomas previously considered to be resistant to bromocriptine and low doses of cabergoline has been proven to be effective. Thus, in the present study, all cases would be considered resistant to cabergoline as defined by these authors, except case 6 who used a maximum dose of 3.0 mg per week of cabergoline, due to side effects with the higher dose.

Most patients with prolactinomas respond to a weekly dose of up to 2 mg of cabergoline, but 15 to 20% of patients may require higher doses to control the tumor (6, 11, 13, 26). When resistance to dopamine agonists is detected, one approach is to increase the dose used. This was the approach used in the cases presented here that used the maximum dose of 3.5 mg per week. This dose was also used in many of the cases described by Vroonen et al. (12). Case 3 used a dose of 3.5 mg for 115 months; subsequently, the dose of cabergoline was increased to 4.5 mg per week, and prolactin concentration decreased but was not normalized (44.57 ng/mL). Other authors have prescribed good results with up to 7.0 mg per week (11, 26, 27). In these cases, echocardiogram monitoring should be performed to evaluate possible cardiac valve impairments (10, 13, 28). In the present study, serial assessments of cardiac valves with Doppler echocardiograms did not show lesions related to prolonged use of high doses of dopamine agonists.

Regarding tumor volume, all patients had a reduction in tumor size; however, the tumor did not disappear, except in case 6 that evolved to primary empty sella syndrome. Case 1, after 14 years of using cabergoline, presented a persistent small lesion with cystic appearance along the left carotid. Case 2, after 17 years of using cabergoline, had a significant reduction in tumor size, but tumor remnants persisted in the sphenoid sinus. Case 3, after 14 years of using dopamine agonists (3 years of using bromocriptine and 11 years of using cabergoline), presented with a small cystic tumor. Case 4, after 11 years of using dopamine agonists and 5 years after transsphenoidal surgery and radiotherapy, had the tumor restricted to the sphenoid sinus. Case 5, after 4 years of treatment with cabergoline, presented with a tumor that was virtually unchanged. Case 6, after 17 years of treatment with dopamine agonists (2 years with bromocriptine and 15 years with cabergoline), was tumor-free and had evolved to secondary empty sella turcica syndrome.

An interesting aspect regarding the decrease in tumor mass is the treatment time for macroprolactinomas (11, 12, 25, 27). To obtain a reduction in tumor mass >50%, 2 years of treatment were required in 95% of 26 patients, even in those who were not considered resistant to the drug (25, 27). Patients in the present study also had to be treated for more than 2 years to achieve a reduction in tumor size.

The disappearance of the tumor in response to cabergoline in cases of resistance is uncommon (19%); however; it is more frequently observed in microadenomas than in macroadenomas. The presence of residual tumor on MRI images at the last assessment was more frequent for macroadenomas (53.3%) than microadenomas (26.7%). In other cases, disappearance of the tumor was not observed in patients resistant to cabergoline (11, 27). It has also been reported that chronic treatment with dopamine agonists would make the tumor less dense than at the start of the treatment (8).

Several options are available for the treatment of patients with prolactinomas resistant to dopamine agonists (7, 9, 10). Four of these were used for the treatment of case 4, which was followed for 11 years: (1) switching from bromocriptine to a more potent agonist, cabergoline; (2) increasing the dose of the agonist above the dose recommended by the manufacturer, i.e., a dose of 3.5 mg per week was used, which is above the recommended dose of 2.0 mg; (3) surgical resection of the tumor was performed 6 years after starting the treatment, and (4) radiotherapy was performed after the surgical treatment.

Replacement of bromocriptine by 1.5 mg per week of cabergoline resulted in an increased prolactin concentration. A high prolactin concentration was maintained using this dose for 4 years; however, images showed a marked decrease in the tumor size. Increasing the dose to 3.5 mg per week for 18 months was not associated with a reduction in prolactin concentration or tumor size. Transsphenoidal surgery was then performed which appears to have contributed to the tumor regression observed on MRI 5 years after the procedure. Surgery and the subsequent tumor regression also caused a significant decrease in prolactin concentration, although it was not normalized. Surgery was also an option for case 5 which presented a normalized prolactin concentration, restored menstrual cycles, and disappearance of the tumor after the transsphenoidal surgery.

Surgery was frequently performed in a previous multicenter study: 60.9% of the patients underwent surgery, especially those with macroadenomas (12). Another likely scenario is that removal of part of the tumor (debulking) may improve the response to cabergoline after surgery. This phenomenon was also described by Vroonen et al. (12) where patients who did not respond to cabergoline had a 50% decrease in the dose of the drug, achieved normalization of prolactin concentrations in 33% of the cases, and showed complete reduction in tumor mass in 36.8% of the cases after surgery. We observed this phenomenon in patient 4 who only achieved control of prolactin concentration and a reduction in tumor size after transsphenoidal surgery. Therefore, surgery may be an alternative treatment for patients resistant to dopamine agonists or for those who do not tolerate the drug (12, 28–30).

The other approach adopted for the treatment of case 4 was radiotherapy with 4,500 rads. Radiotherapy may be useful to control tumor growth but not for normalizing prolactin concentration (8, 10, 12). However, it was observed that after 11 years of treatment with dopamine agonists and 5 years after the surgery and radiotherapy, the tumor had almost disappeared (Figure 8).

Recently, it has been shown that the association of octreotide LAR with cabergoline may improve prolactin concentration and decrease tumor size in some, but not all, cabergoline-resistant prolactinomas (31).

Tumors resistant to dopamine agonists present other characteristics compared to responsive tumors. For example, resistant tumors are more often macroadenomas, more aggressive, invade neighboring structures, are more angiogenic, proliferative, and exhibit cellular atypia (7, 12). The tumors of patients 2 and 4 exhibited these characteristics. In fact, it was suspected that their tumors were not related to the pituitary gland, based on the anomalous growth of the lesions. In general, 17 to 39% of macroadenomas are resistant, while only 4 to 8% of microadenomas are resistant to dopamine agonists (7). In general, microadenomas progress without significant growth, even without treatment, and recurrence after treatment is lower, suggesting that macroadenoma biology is different. Resistant prolactinomas most often present invasion of the cavernous sinus compared to those responding to treatment (11, 12, 32, 33). In the present study, all patients had tumors with these characteristics. In addition, they had a persistent tumor mass several years after starting treatment with the agonist.

Another observation is that macroadenomas resistant to dopamine agonists appear to be more aggressive in men than in women. Complete reduction of tumor size apparently occurs more often in women, even in macroadenomas (11, 12). This was observed in the present study in which the tumors in the two women were less aggressive than those in the four men. One woman (case 6) had a macroprolactinoma with invasion of neighboring structures; over the years of treatment with bromocriptine and later with cabergoline, she presented with tumor regression and eventual evolution to empty sella syndrome.

Genetics may predispose tumors to the development of resistance to cabergoline (12). This possibility was investigated in the present study using patient 3 who presented with a tumor at the age of nine and was a fourth cousin of patient 6. However, assessment of the *AIP* and *NEM1* genes was negative.

Prolactinomas are rare in children (34). In our study, case 3 was diagnosed at the age of nine. During the follow-up, he presented with slow growth. At the age of 15, GH secretion and IGF-1 concentrations were assessed, showing hormonal deficiencies. The patient underwent replacement therapy with GH, and his annual growth rate was satisfactory. At the time, it was hypothesized that treatment with GH could increase the size of the prolactinoma. However, to our knowledge, there is no evidence that exogenous GH can interfere with tumor growth. In fact, during the replacement therapy with this hormone, no increase in tumor size was observed. This case has been published previously (35).

Patient 2 developed psychosis 1 year after a hemorrhagic stroke. For this reason, he started using quetiapine and mirtazapine, which are dopamine antagonists and are associated with increased prolactin concentrations. There was a slight increase in prolactin concentration with the use of these antipsychotics; however, the concentration remained stable until the last assessment, 7 years after starting the psychiatric treatment. Cabergoline, due to its action as a dopamine agonist, can trigger psychiatric disorders and this may be related to the dose used (36). Thus, the dose was lowered to 2.0 mg per week until the last assessment.

Patient 4 had a psychiatric follow-up for severe depression and used drugs for a prolonged period, either together or separately, including escitalopram, alprazolam, pregabalin, and flunitrazepam. However, these treatments did not appear to have influenced the prolactin concentration, as shown in Figure 7. The association of hyperprolactinemia with the use of antidepressants is known to inhibit the reuse of dopamine, which in turn inhibits prolactin (37).

An increased prolactin concentration decreases secretion of FSH and LH by inhibiting hypothalamic GnRH directly in pituitary gonadotrophs. This causes menstrual changes in women and decreased libido in men due to a decreased production of testosterone (38). Thus, many patients require testosterone or estrogen and progesterone replacement to prevent the harmful consequences of hypogonadism, such as osteoporosis, altered lipid control, and glycemia. In addition, a significant increase in tumor size with invasion of neighboring tissues can cause deficiencies in the pituitary hormones, FSH, LH, GH, TSH, and ACTH (38).

Regarding men, case 1 presented panhypopituitarism at the time of diagnosis, which is characterized by gonadotroph, thyrotroph, and corticotroph deficiencies requiring testosterone, levothyroxine, and prednisone replacement therapy. After 1 year, there was recovery of the corticotroph axis, and prednisone was suspended. As in case 1, cases 2 and 4 presented with a deficiency of the gonadotroph axis from the time of diagnosis, and there was no recovery; they required replacement therapy with testosterone throughout the follow-up. In all three these cases, high concentrations of prolactin and giant tumors persisted. It is interesting that case 3 never developed hypogonadism and even had a normal puberty, although always maintaining very high concentrations of prolactin. To our knowledge, this is a unique situation, and we have no explanation for this phenomenon. This case has been published previously (35).

It has been described that testosterone replacement can cause tumor growth and induce resistance to dopamine agonists (39). This is because testosterone is aromatized into estrogen and the latter stimulates the proliferation and hyperplasia of lactotrophs, growth of prolactinomas, and the induction of dopamine resistance (1). In this context, the use of anastrozole, an aromatase inhibitor, was shown to block the increase in prolactin concentration caused by replacement therapy with testosterone in a giant prolactinoma (40). Regarding the three men who underwent replacement therapy with testosterone during the entire follow-up, it appears that this did not interfere with the long-term decrease in the size of their tumors.

Regarding women, case 5 only exhibited normalized menstrual cycles after surgical removal of the prolactinoma. Case 6 had regular menstrual cycles after 28 months of treatment, consisting of 24 months of bromocriptine and four of cabergoline.

Four patients (cases 1, 2, 4, and 6) developed hypothyroidism and underwent replacement therapy with levothyroxine. However, the causes of this alteration varied: cases 1, 2, and 6 developed hypothyroidism secondary to the presence of a macroadenoma in the hypothalamic-pituitary region; case 4 developed primary hypothyroidism secondary to Hashimoto's thyroiditis since the anti-peroxidase antibody was positive at high concentrations, and TSH was elevated. Diagnosis of these two conditions is sometimes difficult; however, it is essential as the follow-up of these patients has certain peculiarities. TSH levels are suitable for the diagnosis of primary hypothyroidism, but not for the diagnosis of central hypothyroidism. This is because TSH may be normal most of the time, is rarely suppressed, or is slightly above the upper normal limit. Lesions in the pituitary-hypothalamic region can lead to the production of immunologically active but biologically inactive TSH (41). In this situation, the best parameter for the diagnosis of central hypothyroidism is the concentration of FT4. Empirically, FT4 is normal if the values are above 1.0 ng/dL and the ideal values during replacement therapy are 1.2 to 1.4 ng/dL (22). Cases 1, 2, and 6 underwent replacement therapy with levothyroxine, maintaining these concentrations of FT4. In case 4, the dose of levothyroxine was adjusted according to TSH levels.

Two patients developed adrenal insufficiency. Case 1 was treated with prednisone for 1 year, but after tumor reduction, normalization of cortisol occurred and persisted throughout follow-up. Case 4 initially had partial cortisol deficiency but did not require replacement. This can occur when there is no significant stress to trigger an adrenal crisis (21). But afterwards, after surgery for the pituitary tumor and radiotherapy, he developed adrenal insufficiency and is on remission with prednisolone (21).

There is a possibility that macroadenomas may be associated with GH deficiencies, as had occurred with patient 3 and is described above. However, in the present study, no other patient had GH deficiencies as analyzed by IGF-1. Assessment of GH secretion through quantification of IGF-1 only may be inconsistent, since the isolated quantification of this hormone does not have diagnostic value for GH deficiencies in adults; however, it may be useful for long-term follow-up (42).

In addition to the known changes in the gonads associated with high concentrations of prolactin, we should also be concerned about the maintenance of high prolactin concentrations that fail to normalize. Many years of high prolactin concentration may have a negative effect on the health of the patient (43). There is increasing interest in the deleterious effects of chronic prolactin excess because hyperprolactinemia has been associated with tumorigenesis in breast cancer in rodents and women (44) as well as prostate cancer (45). Its association with autoimmune diseases, such as systemic lupus erythematosus, rheumatoid arthritis, Sjögren's syndrome, psoriasis, Hashimoto's thyroiditis, multiple sclerosis, Behçet's disease, celiac disease, hepatitis C, and peripartum cardiomyopathies has also been described (46). Interference is described in metabolic homeostasis, regulating key enzymes and carriers associated with lipid and glucose metabolism (47). The association of hyperprolactinemia with increased morbidity and mortality remains controversial. For example, in a population-based study, pituitary tumor-related hyperprolactinemia did not show an increased risk for diabetes, vascular disease, bone fracture, and all causes of cancer and breast cancer; however, macroadenomas, drug-induced hyperprolactinemia, and idiopathic hyperprolactinemia were associated with an increased risk of death (48). Another study showed that in men, but not in women, hyperprolactinemia would be associated with higher all-cause and cardiovascular mortality (49).

Prolactin concentrations remained very high in all patients, even after several years of chronic use of cabergoline. However, we cannot comment on the deleterious effects of hyperprolactinemia in these patients since all manifestations were treated specifically when present: dyslipidemia, diabetes mellitus, and hypertension. We did not observe prostate or breast cancer in this study.

We can conclude from the study of these cases that tumors showing resistance to cabergoline may be more aggressive in men than in women. This study also showed that, although dopamine agonists may not reduce tumor volume by more than 50% in the short term, the use of high doses of cabergoline may reduce tumor volume by more than 90% or promote progression to an empty sella in the long term, even in macroprolactinomas.

Regarding prolactin concentrations, except for one woman who achieved normalized prolactin concentrations after 17 years of treatment, there was no normalization in the remaining five cases. It is possible that even without normalizing prolactin concentrations, a significant decrease is beneficial for these patients. Thus, treatment of prolactinomas with resistance to cabergoline remains a worrying, but there was a significant decrease in prolactin and tumor decrease by more than 90% with the use of high doses of cabergoline in the long term.

This study also showed that, in men, these tumors were diagnosed at an advanced age and incidentally while searching for other diseases.

## Data availability statement

Datasets are available on request: the raw data supporting the conclusions of this manuscript will be made available by the authors, without undue reservation, to any qualified researcher.

## Author contributions

LC, LN, MG, and LdC analyzed the patient data. LC and MG were major contributions in writing the manuscript. JM physician radiologist, analyzed the MRI. BO and IK surgeons, operated on the patients. All authors agreed to be accountable for the content of the work and approved the submitted version of the manuscript.

### Conflict of interest statement

The authors declare that the research was conducted in the absence of any commercial or financial relationships that could be construed as a potential conflict of interest.

## Abbreviations

ACTH: adrenocorticotropic hormone
AMPc, cyclic adenosine monophosphate, FSH: follicle stimulating hormone
GH: growth hormone
IGF: insulin-like growth factor-1
LH: luteinizing hormone
MRI: magnetic resonance imaging
FT4: free thyroxine
TSH: thyroid stimulating hormone.
